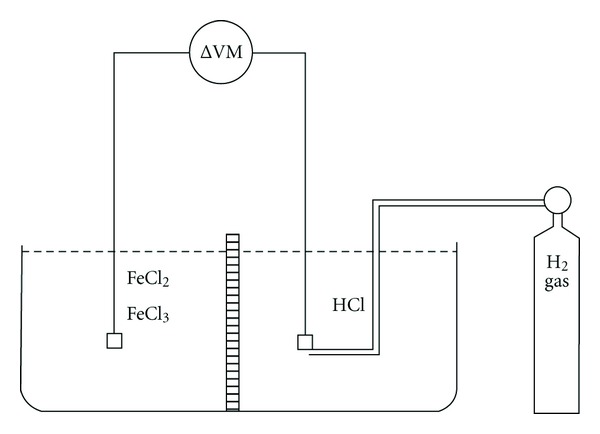# The Confluence of Faraday's and Kirchoff's Laws in Bioelectrochemical Systems

**DOI:** 10.1100/2012/838756

**Published:** 2012-04-19

**Authors:** Harvey N. Seiger

**Affiliations:** Naval Systems Division, Electrochemical Science and Technology Associates, Boynton Beach, FL 33437, USA

## Abstract

When external measurements are made of electrochemical systems, including bioelectrochemical, there results an interaction. Such measurements cause electrochemical processes to take place that are significant. This work looks into the nature and significance of the interfacial processes on membrane and membrane phenomena. The conclusion reached is that interfacial processes are important and cannot be overlooked.

## 1. Introduction

Faraday's and Kirchoff's Laws are applied to electrochemical systems that have two compartments separated by a salt bridge or a membrane. The act making voltage measurements results in a closing of the circuit. There are processes that occur at the solution/metal interfaces that cannot be neglected. Ignoring these interfacial processes result in obfuscated conclusions.

Faraday's Law defines ions as the traveler in the electrolyte when current is flowing. He further defines the anode as the electrode at which oxidation occurs and the cathode at which reduction occurs. As a corollary, when the system is placed on open circuit, since oxidation-reduction reactions are not occurring, there is only a conductor dipping in an electrolyte solution. Recognizing that, colloquially, a combination of a conductor dipping an electrolytic solution, say FeCl_2_ and FeCl_3_, is referred to as an electrode, it is not an electrode that fits the definition but should better be termed a preelectrode because the circuit is not completed.

Consider the electrochemical circuit of Figure 1. It features two compartments that may be separated by an agar salt bridge, an asbestos thread, a semipermeable membrane, or a fritted glass disk. Let us have a solution of FeCl_3_ and FeCl_2_ in one compartment with a platinum wire inserted into this compartment and a standard hydrogen electrode (SHE) in the second compartment. The electrode reactions may be written as (when connected at least through a meter).

Anode: 1/2H_2_→H^+^ + e^−^

Cathode: Fe^+3^ + e^−^→Fe^+2^

Recognizing that we are dealing with the chlorides, these equations may be written as equivalently as follows.

Anode: 1/2H_2_ + Cl^−^→HCl + e^−^

Cathode: Fe^+3^ + 2Cl^−^ + e^−^→FeCl_2_

The first form emphasizes the charge transfer processes between the solutions and conductors dipping into them. The second form emphasizes the ionic conduction between the compartments.

It is noted that the current in this closed circuit may be measured by having an external meter in the electronic pathway or by making use of Ampere's Law to measure the magnetic field induced by the current flow. This measurement can be made using magnetometers such as “clamp on” meters [1] or the more esoteric SQUID (for superconducting quantum interference device) without breaking the circuit. Actually, if one believes that some current flows in an open circuit system, these devices may be used for confirmation. 

Kirchoff's Law becomes important in bioelectrochemical systems where a “ground” electrode is also in contact with the electrolyte where the current path branches.

## 2. Nernst Equation

The Nernst equation is derivable from the Gibbs Free Energy starting with

(1) $$\Delta G^{\text{o}}=-\;{zFE}_{\text{cell}}^{\text{o}},$$

where Δ*G* ^*o*^ is the Gibbs Free Energy, *z* is the number of electrons involved in the electron transfer at the electrode interfaces, *F* is the Faraday constant, and *E*_cell_^o^ is the voltage measured at the terminals.

Nenrst noted that when not at standard conditions,

(2) $$E_{\text{cell}\;}=E_{\text{cell}}^{\text{o}}-\left(\frac{\text{RT}}{zF}\right)\text{lnQ},$$

where Q is the reaction quotient based on actual concentrations. The value of *E*_cell_ is measured at the terminals. Remember that such a measurement results in a closed circuit and there is no known way to measure a single electrode, only a completed circuit. This is entirely different than dealing with electrostatic charge where there are electrostatic meters. If there is discontinuity, then, without the oxidation-reduction reaction, these become merely conductors dipping into electrolytic solutions and there is no electrochemistry.

As a consequence, oxidation-reduction potentials are arbitrarily assigned the value of zero to the SHE and potentials are measured from the electrode of interest to the SHE.

At this point, it is of interest to know exactly what electrode process is occurring. Let us consider three cases when this becomes important. The first case is the hydrogen-oxygen system, commonly recognized as the fuel cell proposed for electric vehicle propulsion. According to the standard oxidation-reduction potential, this electrochemical cell should read 1.229 volts on open circuit. The highest voltage experimentally observed is about 1.05 volts. The speculation is that the initial product of the reduction of oxygen is H_2_O_2_ and not H_2_O.

The second example is the case of, say, Fe^+2^/Fe^+3^ versus the SHE. With both at unit activity, the potential is 0.077 volts. Using the Nernst equation and decreasing the activities of the ions, Fe^+2^ and Fe^+3^, while not changing the other chamber of Figure 1, the calculated potential using the Nernst equation is still 0.077 volts. However, the chamber of one side of Figure 1 contains the unit activity ratio of Fe^2+^/Fe^+3^, and the other chamber has the same ratio of Fe^+2^/Fe^+3^ at 0.1 unit activity, then the system is a concentration cell and delivers 59 mV. The electrode reactions for the concentration cell differ from the iron/hydrogen system.

The third example occurs with significant current impressed upon the system [2]. The overall system in one in which nickel hydroxide is deposited from an acidic nickel nitrate solution into the pores of a sintered nickel by cathodization. The counter electrode reaction is just the electrolysis of water. The overall reaction is as follows:

(3) $$\text{HN}{\text{O}}_{3}+2{\text{H}}_{2}\to {\text{NH}}_{4}\text{OH}+2{\text{O}}_{2}$$

The ammonia is produced within the pores of the cathode while oxygen is generated at the anode. The ammonia production results in precipitation of the Ni(OH)_2_ resulting in impregnation that is represented by the following equation:

(4) $$\left(9-x\right){\text{Ni}}^{+2}+2\left(9-x\right){\text{OH}}^{-}\to \left(9-x\right)\text{Ni}{\left(\text{OH}\right)}_{2}$$

The value of *x* depends upon current density. This demonstrates that the processes at the electrode change may be dependent upon current density at which polarization is made to take place.

These three examples indicate, again, the need to know the electrode processes that take place when the circuit is closed, by even a meter, thereby making an electrochemical system.

## 3. Loeb's Direct Measurement of “Membrane Potentials”

Prior to Loeb's [3] work in 1922, systems were measured using two SHEs. Loeb used a collodion bag as the separating membrane in Figure 1. He had a gelatin chloride in one compartment and an HCl solution in the other. Analyzing, first, Loeb's measurements using an electrometer having a high impedance, potential differences were measured when he used calomel electrodes.

He calculated the potential differences between the two compartments as he changed the concentration, later changed the anions, and found there was an agreement between measurements of the membrane potential (see Loeb's Tables XI XII in his chapter on Membrane Potentials).

The calomel reference electrodes consist of a copper wire dipping into elemental mercury. The mercury is in contact with calomel (Hg_2_Cl_2_) which is bathed by the electrolyte in that compartment. When the circuit is closed, by a load, or a meter, or both in parallel, Hg is oxidized to calomel in the anode compartment:

(5) $${\text{Hg}}^{\text{o}}\to {\text{Hg}}^{+}+{\text{e}}^{-}$$

but

(6) $${\text{Hg}}^{+}+{\text{Cl}}^{-}\to 1/2{\text{Hg}}_{2}{\text{Cl}}_{2}$$

The electron effectively travels through the eternal circuit to the other charge transfer interface where it reaches the calomel and

(7) $${\text{e}}^{-}+1/2{\text{Hg}}_{2}{\text{Cl}}_{2}\to 1/2{\text{H}}_{2}+{\text{Cl}}^{-}$$

thereby increasing the concentration of Cl^−^ ions. These Cl^−^ ions migrate through the membrane to the other compartment.

Thusly, there is electronic flow in the external circuit to reach the cathode region where there is an electronic transfer resulting in the reduction reaction. Similarly, the Cl^−^ ion flows through the electrolyte of the cathode compartment, through the separating membrane, through the electrolyte in the anode compartment, where it completes the circuit. The charge transfer rates of the two electrodes, the electronic transfer rates, and finally the ionic fluxes are all the same as required by Kirchoff's law. Note, however, the precipitation of Hg_2_Cl_2_ in one compartment and the consumption of Hg_2_Cl_2_ in the other compartment. Thus, there is a mechanism for concentration changes in both compartments.

Loeb believed that he was measuring the membrane potential directly. Now, we can see that he was measuring the electrode process that can occur using calomel, or the other popular reference electrode:

(8) Ag,Ag//HCl

Since, this too contains a mechanism for concentration changes in both compartments.

Now, consider the analysis of the SHE where gaseous hydrogen is bubbling over a platinum surface immersed in an aqueous HCl solution. There is no mechanism for concentration changes in the separate compartments. As a result, there is no electrochemical reaction and no detectable voltages of significance. Thus, again, it is found that electrode process matters and that the expected voltage using the SHE results in an assignment of “membrane potential” to a situation depending upon the nature of the related reference electrodes.

## 4. The Goldman Equation

Investigating the electrical properties of membranes, Goldman [4] assumed a potential in the solution at point *x*. The ensuing derivation results in a equation for a flux, i(V), caused by a potential difference. Using Ampere's law, one should be able to determine the flux of ions while the system is on open circuit (references not connected through a load or meter). Such measurements have not been found in relevant work. If a question is raised about the application of Ampere's law to determine fluxes of ions, a similar measurement was found earlier [1].

## 5. Conclusions

Electrochemical systems involve oxidation-reduction reactions that are inseparable, the oxidative part at one interface termed, by Faraday, the anode, and the reduction of an equivalent amount at the other interface. Kirchoff's law requires that the rate of electron transfer reactions at the two interfaces be equivalent and that the rate at which the electrons flow (the current) be equivalent as well as the flux of ions. Thus, to have a consistent approach, the processes at the interfaces are important.

Jacques Loeb [3] did not consider the processes at the electrodes when he made the Nernst equation calculations. He overlooked the important interfacial processes. Similarly, Goldman [4] neglects the interfacial reactions. The use of the Nernst equation requires a set of charge transfer interfaces even though they do not appear in the equation.

## Figures and Tables

**Figure 1:**